# Abaloparatide effects on BMD in acetabular regions corresponding to DeLee and Charnley zones in women with postmenopausal osteoporosis

**DOI:** 10.1016/j.bonr.2025.101858

**Published:** 2025-07-19

**Authors:** Neil P. Sheth, Kelly Krohn, Jared Torkelson, Renaud Winzenrieth, Ludovic Humbert, Leny Pearman, Yamei Wang, John I. Boxberger, Mathias P. Bostrom

**Affiliations:** a Department of Orthopaedic Surgery, University of Pennsylvania Hospital- Cathcart Building, 800 Spruce St, Philadelphia, PA 19107, USA; b Department of Orthopedic Surgery, University of Arizona Phoenix College of Medicine, Phoenix, AZ 85004, USA; c Orthopedic Surgery and Sports Medicine, Mayo Clinic Health System, 1025 Marsh St, Mankato, MN 56001, USA; d 3D-Shaper Medical, Rambla Catalunya 53, 4°-H, 08007 Barcelona, Spain; e Medical Affairs, Radius Health Inc., 22 Boston Wharf Rd., 7th Fl, Boston, MA 00210, USA; f Business and Corporate Development, Radius Health Inc., 22 Boston Wharf Rd., 7th Fl, Boston, MA 00210, USA; g Adult Reconstruction & Joint Replacement, Hospital for Special Surgery, 535 East 70th Street, New York, NY 10021, USA

**Keywords:** Abaloparatide, Osteoporosis, Acetabulum, DXA, Bone loss

## Abstract

**Background:**

Acetabular bone loss in patients with osteoporosis is associated with increased risk of acetabular fragility fractures, significant morbidity, and can increase risk of complications in patients undergoing total hip arthroplasty. The anabolic osteoporosis treatment abaloparatide increases total hip areal bone mineral density (BMD), but its effect on acetabular BMD is unknown.

**Methods:**

Anatomical landmarks were identified in DXA scans from a random subgroup of postmenopausal women with osteoporosis (PMO) treated with abaloparatide 80 μg/d or placebo (n = 250/group) from the phase 3 ACTIVE trial to virtually place a hemispherical shell model of an acetabular cup and define regions of interest corresponding to DeLee and Charnley zones 1 (R1), 2 (R2), and 3 (R3). Changes in BMD from baseline at 6 and 18 months were calculated. Statistical significance was tested using a mixed model with repeated measures. Local mean changes in BMD were depicted by alignment of DXA scans via intensity-based registration onto a reference scan.

**Results:**

Abaloparatide significantly increased acetabular areal BMD in all three DeLee and Charnley zones at 6 and 18 months versus placebo. Mean BMD increases with abaloparatide were 8.38 % (R1), 7.25 % (R2), and 9.73 % (R3) at 18 months. BMD increases were homogenously distributed throughout the regions. With placebo, localized losses in BMD were noted after 18 months.

**Conclusions:**

Abaloparatide treatment rapidly and progressively increases BMD in acetabular zones in PMO.

**Clinical trial number:**

NCT01343004.

## Highlights

• Abaloparatide showed beneficial effects on acetabular bone.
• Significant BMD effects were seen in DeLee and Charnley zones at 6 and 18 months.
• These findings may have implications for reducing fractures of the acetabulum.
• Increasing acetabular BMD is an attractive goal for reducing complications in THA.
• Further studies are warranted to investigate these effects on acetabular bone.

## Graphical abstract

**Figure:**
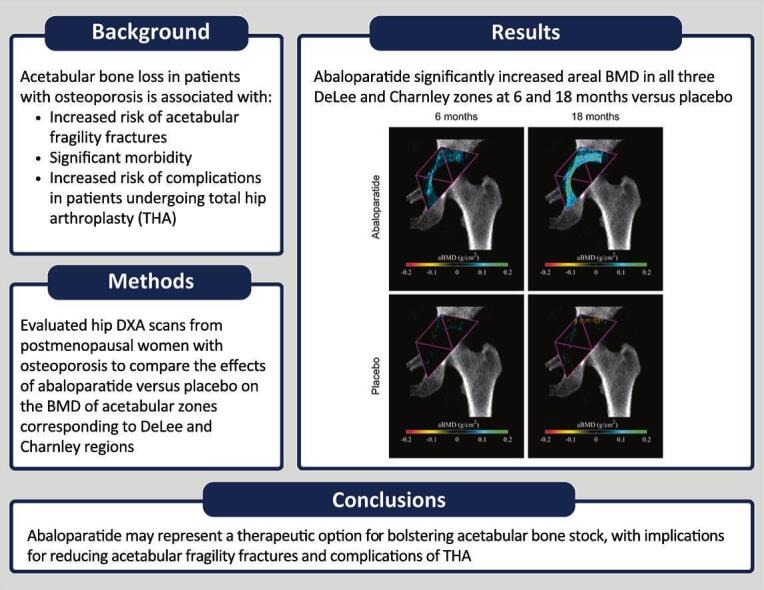

## Glossary

aBMD: Areal BMD
ACTIVE: Abaloparatide Comparator Trial in Vertebral Endpoints
BHO: Bone health optimization
BMD: Bone mineral density
BMI: Body mass index
DXA: Dual-energy X-ray absorptiometry
FN: Femoral neck
LS: Lumbar spine
PTHrP: Parathyroid hormone-related protein
RANKL: Receptor activator of nuclear factor-κB ligand
TH: Total hip
THA: Total hip arthroplasty

## 1 Introduction

Millions of patients with osteoporosis experience fragility fractures every year (Johnell and Kanis, 2006). Fifty percent of women and 25 % of men over the age of 50 will experience an osteoporotic fracture (National Osteoporosis Foundation, 2011, Office of the Surgeon General (US), 2004). Though osteoporotic fractures are most commonly associated with the spine, hip, and forearm, the loss of bone mineral density (BMD) and compromised bone structure occurs throughout the skeleton (Johnell and Kanis, 2006, Office of the Surgeon General (US), 2004). Among sites prone to bone loss with age is the acetabulum (Hauser et al., 1997), with downstream sequelae of reduced acetabular BMD, including fragility fracture, and complications associated with total hip arthroplasty (THA) (Anderl et al., 2020; Firoozabadi et al., 2017; Khakpour et al., 2022; Walls et al., 2021). Decreasing BMD loss or increasing acetabular BMD are attractive goals in osteoporotic patients prone to fracture, many of whom will require THA.

Osteoporosis significantly alters bone structure and reduces BMD, resulting in compromised strength at affected skeletal sites (Cosman et al., 2014). Acetabular bone is not immune to the effects of osteoporosis, and failure is substantially affected by the reduction in the mechanical properties of trabecular and cortical bone (Khakpour et al., 2022). As life expectancy continues to increase, prevalence of acetabular fractures is increasing. There was a reported 2.4-fold increase between 1980 and 2007 in those aged ≥60 years (Ferguson et al., 2010). The overall rate of increase since then is unclear, but is increasing with age (Khalifa et al., 2024; Zhu et al., 2019). Upwards of 12.4 % of all acetabular fractures are a result of a low-energy mechanism (Ferguson et al., 2010; Papadakos et al., 2014), with most occurring due to falls from a standing height (Firoozabadi et al., 2017; Papadakos et al., 2014). The greater reduction in mechanical properties seen with advanced osteoporosis can be associated with more complex fracture patterns, which often require surgical intervention and rehabilitation (Khakpour et al., 2022). Treatments which improve acetabular BMD and reduce acetabular fracture incidence would reduce clinical burden.

In addition to an increase in fragility fractures, low BMD contributes to an increased risk of implant-associated complications (Finnilä et al., 2016). Periprosthetic acetabular BMD loss after THA is a known procedural consequence (Digas et al., 2006; Driscoll et al., 2023; Lazarinis et al., 2013), further compromising patients with low BMD at baseline. Several studies have connected low BMD with an increased risk of revision surgeries. Among patients undergoing THA, patients with low versus normal BMD were at higher risk for revision by 67 % and 20 %, respectively (Finnilä et al., 2016). Acetabular revision is a limiting factor in implant longevity (Patel et al., 2023; Upfill-Brown et al., 2021); revision surgery is challenging when managing substantial acetabular bone loss (Pakvis et al., 2016; Shon et al., 2016). Improved BMD and a potential reduction in THA failure and subsequent complications is critical for implant longevity. Surgical or pharmacological approaches accomplishing this goal need to be evaluated.

Abaloparatide, a synthetic 34-amino acid peptide with homology to human parathyroid hormone-related protein (PTHrP), favors bone formation by selective activation of PTH receptor type 1 (Hattersley et al., 2016). Data from the phase 3 Abaloparatide Comparator Trial in Vertebral Endpoints (ACTIVE, NCT01343004) study in postmenopausal women with osteoporosis showed that abaloparatide significantly increased areal BMD (aBMD) versus placebo at the femoral neck, total hip, and lumbar spine after 6, 12, and 18 months of treatment (Miller et al., 2016). Abaloparatide significantly increased BMD compared with open-label teriparatide at the total hip and femoral neck at all three timepoints and at the lumbar spine at 6 and 12 months. Iliac crest bone biopsies of participants in the ACTIVE study further revealed higher cortical porosity in both the abaloparatide- and teriparatide-treated groups, as well as normal bone microarchitecture and a lower amount of eroded surface in the abaloparatide-treated group.(Moreira et al., 2017) Post hoc analysis of the ACTIVE data showed that abaloparatide increased cortical and trabecular volumetric BMD at the proximal femur versus placebo (Winzenrieth et al., 2021). The effects of abaloparatide on the femur and spine are well established (Miller et al., 2016; Winzenrieth et al., 2021); however, the effect on the acetabulum remains unknown. The current study evaluates hip DXA scans from postmenopausal women with osteoporosis to compare the effects of abaloparatide versus placebo on the BMD of acetabular zones corresponding to DeLee and Charnley regions.

## 2 Methods

### 2.1 Study design

The ACTIVE trial was a phase 3, double-blind, placebo-controlled trial comparing the efficacy and safety of abaloparatide versus placebo, with an additional open-label teriparatide arm (Miller et al., 2016). The trial enrolled 2463 postmenopausal women, aged 49 to 86 years, with BMD T-scores ≤−2.5 and >−5.0 at the femoral neck (FN) or lumbar spine (LS) and had either ≥2 mild or 1 moderate vertebral fracture or a nonvertebral fracture within the past 5 years (Miller et al., 2016). Patients aged >65 years were included with the above fracture criteria and a BMD T-score ≤−2.0 and >−5.0 at either site, or without meeting fracture criteria with a BMD T-score ≤−3.0 and >−5.0 (Miller et al., 2016). Full entry and exclusion criteria have been previously described (Miller et al., 2016). Patients were randomized 1:1:1 to receive daily subcutaneous injections of abaloparatide (80 μg/day), teriparatide (20 μg/day), or placebo (Miller et al., 2016). DXA scans were collected at the spine and hip at baseline, 6, 12, and 18 months (Miller et al., 2016).

For the current study, a subset of 500 patients (abaloparatide, n = 250; placebo, n = 250) from the ACTIVE trial were randomly selected with stratification by study site and patient race/ethnicity to ensure uniformity across groups for analysis of acetabular BMD (Winzenrieth et al., 2021). Patient DXA scans were evaluated at baseline, 6 months, and 18 months.

The ACTIVE study was approved by the ethics committee at every participating institution and was conducted according to Good Clinical Practice recommendations and the Declaration of Helsinki (Miller et al., 2016). All participating patients provided written informed consent (Miller et al., 2016).

### 2.2 DeLee and Charnley zones analysis

**Fig. 1:**
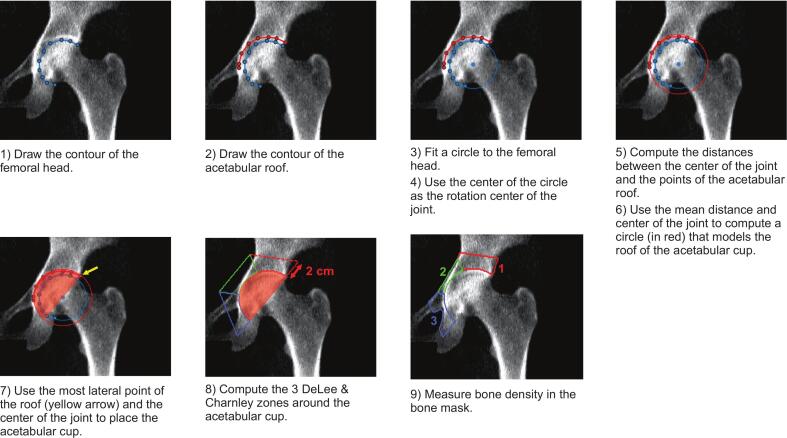
Workflow of virtual placement of the acetabular cup and calculation of DeLee and Charnley zones.

To quantify regional BMD changes in the acetabulum, DeLee and Charnley (Anderson et al., 2019; DeLee and Charnley, 1976) zones were defined within each DXA image using a semiautomated procedure (Fig. 1). First, the contour of the femoral head and the contour of the acetabular roof were manually defined. Next, a circle was automatically fitted to the drawn femoral head contour using the least squares method. The center of the fit circle was taken as the rotational center of the joint. Next, distances between the drawn acetabular roof and the rotational center were calculated and the mean distance computed. A second circle was then created using the rotational center as the origin and the mean distance to the acetabular roof as the radius. This circle defined the acetabular roof for modeling purposes. Next, the most lateral point of the acetabular roof was used, and a line was passed through this point and the rotational center. This line, along with a circle fitted to the acetabular roof, was used to define the boundary of a hemispherical cup, which was virtually placed in the DXA image, mimicking the shell of Styker's Trident PSL Acetabular Cup.

DeLee and Charnley zones were calculated based upon the original definition by DeLee and Charnley, while including adaptations for determination via hip DXA scans (Anderson et al., 2019; DeLee and Charnley, 1976). To do so, wedges were created by dividing the hemispherical acetabular cup into thirds and extending lines outward from the center of the acetabulum. Zones were extended a distance of 2 cm from the cup periphery. Within the defined DeLee and Charnley zones, bone region was automatically identified using thresholding methods, and the mean bone density was quantified within each region.

### 2.3 Data analyses

Statistical analyses were performed with *P* values derived from contrast tests based on a mixed model with repeated measures (Winzenrieth et al., 2021). The model was adjusted for body mass index (BMI), age, and BMD value at baseline (Winzenrieth et al., 2021). Other covariates included DXA scanner type (Winzenrieth et al., 2021), treatment group, visit, and treatment group/visit interaction (Winzenrieth et al., 2022).

A secondary analysis was performed to enable visualization of the distribution of aBMD changes. Intensity-based nonrigid registration methods were utilized to align and scale all DXA scans from the database onto a reference DXA image. Differences between the aligned DXA images at 6 months and 18 months versus baseline were calculated. Average differences at each time point were calculated. The spatial distribution of the averages of the aBMD changes were shown for both the abaloparatide and placebo groups using a color map.

## 3 Results

Baseline characteristics of the subset of patients (N = 500) included in this analysis were well balanced and previously described (Winzenrieth et al., 2021). Briefly, mean (SD) age was 69 (6.1) in the placebo group and 69 (6.7) in the abaloparatide group and 79 % were White in both groups. Acetabular aBMD was matched between the two groups at baseline, except for zone 1, where it was greater in the placebo group, with aBMD baseline comparison *P* values of 0.041, 0.465, and 0.305 for zones 1, 2, and 3, respectively (Table 1).

**Table 1:** Baseline areal BMD.

	Baseline (g/cm^2^)		*P* value^a^
	ABL (N = 250)	PBO (N = 250)	
Zone 1	1.14 (1.11, 1.16)	1.17 (1.15, 1.19)	0.041
Zone 2	1.01 (0.98, 1.03)	0.99 (0.97, 1.02)	0.465
Zone 3	0.80 (0.78, 0.82)	0.81 (0.79, 0.83)	0.305

ABL, abaloparatide; BMD, bone mineral density; CI, confidence interval; PBO, placebo.

Values presented as mean (95 % CI).

a Areal BMD at baseline comparison *P* values are derived using student *t*-test.

**Fig. 2:**
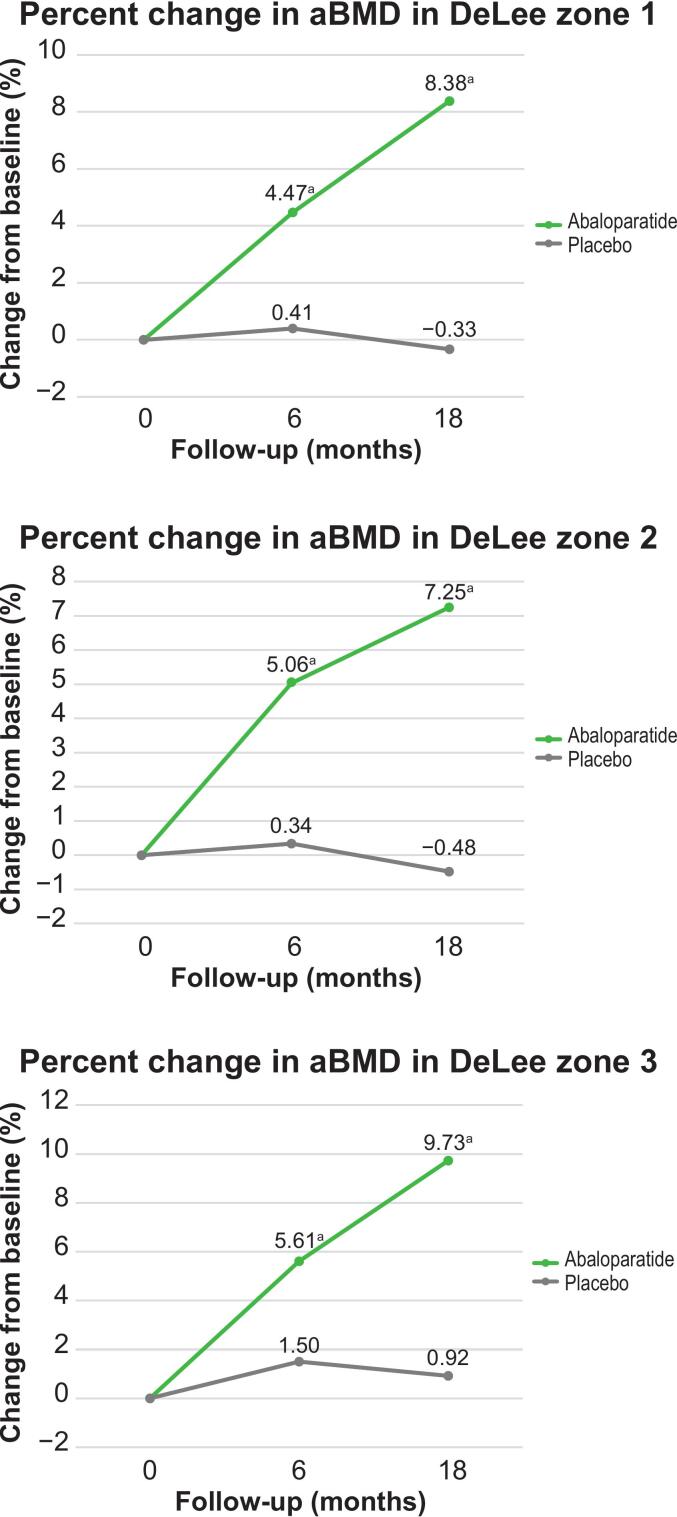
**Mean percent change from baseline in aBMD in DeLee zones 1, 2, and 3.** aBMD, areal bone mineral density. ^a^*P* value <0.001.

Areal BMD significantly increased from baseline in all DeLee and Charnley zones with abaloparatide treatment versus placebo at months 6 and 18 (all *P* < 0.0001 vs placebo) (Table 2; Fig. 2). In patients receiving placebo, a decrease in aBMD was observed in zones 1 and 2 at 18 months, with a larger decrease in zone 2.

**Table 2:** Mean percent change from baseline in aBMD in DeLee zones 1, 2, and 3 at 6 and 18 months.

	Mean change from baseline (%)		*P* value^a^
	ABL (N = 250)	PBO (N = 250)	
Zone 1			
6 months	4.47	0.41	<0.0001
18 months	8.38	−0.33	<0.0001
Zone 2			
6 months	5.06	0.34	<0.0001
18 months	7.25	−0.48	<0.0001
Zone 3			
6 months	5.61	1.50	<0.0001
18 months	9.73	0.92	<0.0001

ABL, abaloparatide; aBMD, areal bone mineral density; BMI, body mass index; DXA, dual-energy X-ray absorptiometry; MMRM, mixed model for repeated measures; PBO, placebo.

a *P* values were derived from contrast tests based on an MMRM model fitted using only the data of the two treatment groups to be compared. Model adjusted for BMI, age, and value at baseline; other covariates including DXA scanner type, treatment group, visit, and visit interactions.

**Fig. 3:**
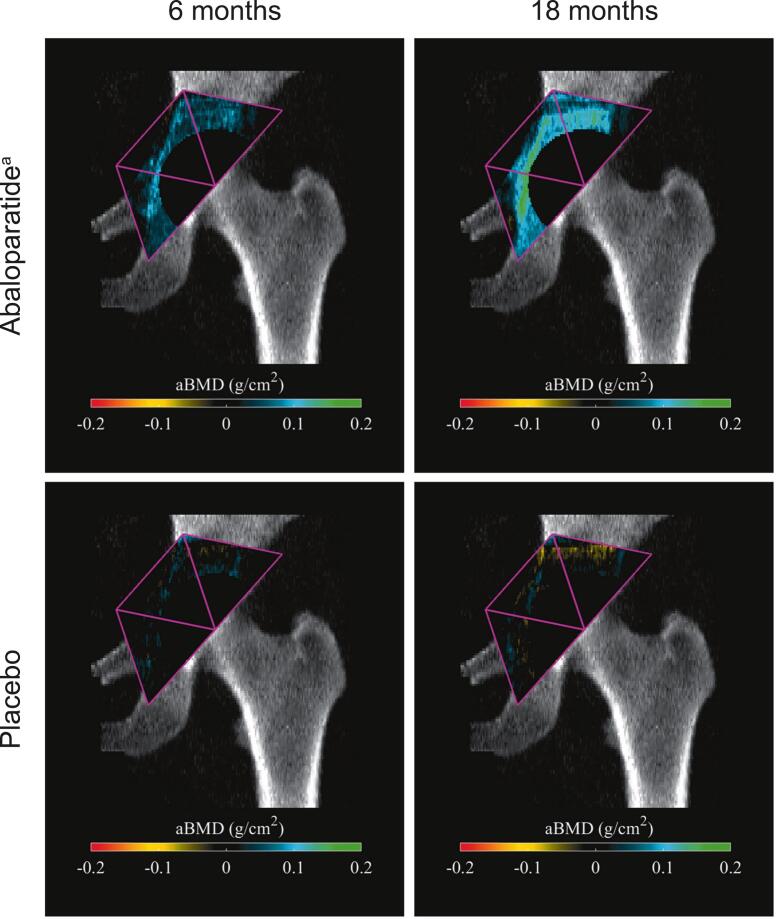
Color maps of the spatial distribution of mean aBMD changes from baseline aBMD, areal bone mineral density. ^a^*P* value <0.001 for aBMD absolute change from baseline at 6 and 18 months in DeLee zones 1, 2, and 3.

Color maps of the spatial distribution of mean aBMD changes from baseline in the abaloparatide and placebo groups at 6 and 18 months are shown in Fig. 3. With abaloparatide treatment, consistent gains in aBMD are well distributed within the three zones at 6 months. In patients receiving placebo, absolute aBMD remains largely unchanged from baseline at 6 months with some local small increases and decreases noted, while at 18 months, more localized decreases in aBMD are noted, particularly in the area superior to where the acetabular cup would be placed, largely corresponding to zone 1.

## 4 Discussion

In the current study, abaloparatide significantly increased aBMD in all three DeLee and Charnley regions at both 6 and 18 months versus placebo. The increase of BMD was well distributed throughout the regions, while with placebo, localized losses in BMD occurred after 18 months.

The finding of increased BMD in the acetabular regions at 6 and 18 months is likely clinically significant, in addition to being statistically significant. The increase in BMD in all zones was approximately 5 % by 6 months and approaching 10 % in zone 3 by 18 months. These results are consistent with the effects of abaloparatide in the trabecular-rich LS, where abaloparatide significantly increased aBMD by 6.6 % and 11.2 % at 6 and 18 months, respectively, for both the full ACTIVE population and the 250 patient subgroups from the current study (all *P* < 0.001 vs placebo) (Miller et al., 2016). The BMD results here in the acetabular regions exceeded BMD gains in the total hip (TH), where increases with abaloparatide treatment in TH BMD of 2.32 % at 6 months and 4.18 % at 18 months were observed (Miller et al., 2016). In a previous study involving this same subset of ACTIVE patients, abaloparatide treatment resulted in a significant increase of 4.2 % in TH aBMD and approximately 9 % in trabecular volumetric BMD at the proximal femur at 18 months (both *P* < 0.001 vs placebo) (Winzenrieth et al., 2021). Increases in FN BMD of 1.72 % at 6 months and 3.60 % at 18 months were also seen with abaloparatide treatment in ACTIVE (Miller et al., 2016). The BMD gains in the LS, FN, and TH are factors contributing to an observed reduction in vertebral fracture and nonvertebral fracture risk at 18 months for abaloparatide (Miller et al., 2016), reinforcing the clinical importance of the magnitude of BMD change. Indeed, reduction in the mechanical properties of cortical and trabecular bone has been linked to acetabular failure previously (Khakpour et al., 2022).

Increased acetabular BMD may have beneficial implications for THA outcomes. The scenario is akin to a BHO platform, where BMD is improved prior to THA. The anabolic nature of abaloparatide may prove advantageous given the more rapid accrual of BMD at the hip and spine relative to antiresorptive agents (Hadji et al., 2012; Saag et al., 2017, Saag et al., 2007). Iliac crest biopsies for various drug mechanisms of action have demonstrated increased and accelerated bone formation with anabolics versus antiresorptives (Dempster et al., 2016, Dempster et al., 2018). The 6-month time point may represent a realistic clinical scenario striking a balance between elective surgery urgency and benefit of BMD accrual, while the 18-month time point may represent the fortuitous benefit of patients treated for osteoporosis who may unknowingly require a THA in the future. Acetabular zones have been shown to lose significant amounts of bone following THA (Kiritopoulos et al., 2022). Boosting the starting acetabular BMD may ultimately provide an increased buffer for losing BMD after arthroplasty. Improvement in aBMD and trabecular bone could potentially improve the quality of the contact surface between bone and the acetabular cup, resulting in a better press-fit and distribution of the strain over the acetabular cup. Pitto et al. observed marked periacetabular cancellous bone loss, up to −34 %, though loss in cortical bone was more moderate with various press-fit cup components after 1 year of follow-up (Pitto et al., 2008). Penny et al. demonstrated reduced BMD on the acetabular side of the THA from 4.9 % to 9.1 % depending on the specific zone through 2 years of study (Penny et al., 2012). Pakvis et al. also found a decrease (−14 % to −35 %) in cancellous BMD, yet fairly stable cortical BMD after 2 years following implantation of an elastic press-fit cup (Pakvis et al., 2016). A meta-analysis by Robertson et al. confirmed periacetabular bone loss after THA (Robertson et al., 2023). Future studies should evaluate the paradigm of peri- or postoperative abaloparatide treatment, as is being investigated currently in total knee arthroplasty (NCT04167163).

Other agents have been evaluated in studies of acetabular BMD (Kiritopoulos et al., 2022; Wilkinson et al., 2005, Wilkinson et al., 2001), though treatment was given after the THA procedure, different from our modeled scenario where acetabular BMD effects of abaloparatide were evaluated in unimplanted hips as in the scenario of BHO. Pamidronate, a bisphosphonate, was investigated for its effect on pelvic bone after THA (Wilkinson et al., 2005, Wilkinson et al., 2001). While pamidronate reduced femoral bone loss in the femoral calcar region, it did not affect pelvic bone loss or acetabular cup migration (Wilkinson et al., 2005). A retrospective analysis of a US administrative claims database found that patients with a prior diagnosis of hip osteoarthritis and osteoporosis/osteopenia who were treated with oral and IV bisphosphonates for at least 1 year prior to THA were associated with higher rates of intraoperative and postoperative complications (Jeong et al., 2023). Conversely, a systematic review found that bisphosphonate use for up to 1 year after total joint arthroplasty may decrease postoperative BMD loss and increase the longevity of implants (McDonald et al., 2022). Denosumab was also evaluated for an effect on acetabular bone around an uncemented cup after THA (Kiritopoulos et al., 2022). After 12 months, denosumab-treated patients had higher acetabular BMD versus placebo-treated patients, though after discontinuation and by 24 months the effects declined and turnover markers returned to normal (Kiritopoulos et al., 2022). This is a known consequence of denosumab therapy discontinuation, and a longer treatment course or change to alternative therapy is likely required. Regardless, the evidence suggests that an antiresorptive mechanism, in this case, receptor activator of nuclear factor-κB ligand (RANKL) inhibition, may be effective at mitigating acetabular bone loss. This differs from the mechanism of abaloparatide, which acts to increase bone formation through increased bone remodeling and modeling (Dempster et al., 2021; Hattersley et al., 2016), and ultimately may provide an alternative approach targeting the formation of new bone at the acetabulum. A head-to-head trial comparing different mechanisms of action would be fruitful in furthering the understanding of preventing postoperative bone loss and reducing downstream complications.

The current study, which is suggestive of a beneficial effect of abaloparatide on acetabular BMD and a potential role in BHO, is not without limitations. Due to the nature of the ACTIVE study, patients suffering from osteoporosis were not candidates for elective THA, and the findings reported here may not be generalizable to patients undergoing elective THA, who typically have osteoarthritis. It is possible that the effects of abaloparatide may differ in a different demographic, though past results have suggested that abaloparatide is equally effective in patients with varying demographics (i.e., age, baseline BMD, prior fracture history) (Cosman et al., 2017). The current study was limited to women, and it is unclear if the effects of abaloparatide on acetabular BMD would be the same in men. Notably, BMD effects at other skeletal sites were consistent between men and women through twelve months of treatment (Czerwinski et al., 2022; Miller et al., 2016). Results may have been slightly different through utilization of a different modeled acetabular cup geometry or through use of three-dimensional imaging (as opposed to two-dimensional DXA), though the approach here permitted a consistent technique across all patient scans and represents the standard technique for quantifying BMD at other common skeletal sites in studies and patient care.

## 5 Conclusions

The current study is suggestive of beneficial effects of abaloparatide in acetabular bone, specifically in regions surrounding a (theoretical) acetabular cup. These findings may have implications for reducing both fragility fractures of the acetabulum and complications attributable to acetabular cup failure associated with reduced BMD in THA. The results support the recommendation of Kadri et al. (2020), that anabolic therapy such as abaloparatide be considered as an option for BHO, and suggest that benefits may extend beyond the traditionally considered skeletal sites of the femur and spine. Further studies are warranted to more deeply investigate the effects of pre-, peri-, and postoperative abaloparatide treatment on acetabular bone and acetabular cup component longevity.

## CRediT authorship contribution statement

**Neil P. Sheth:** Writing – review & editing, Data curation, Conceptualization. **Kelly Krohn:** Writing – review & editing, Data curation. **Jared Torkelson:** Writing – review & editing, Data curation. **Renaud Winzenrieth:** Writing – review & editing, Software, Methodology, Formal analysis, Data curation, Conceptualization. **Ludovic Humbert:** Writing – review & editing, Writing – original draft, Software, Methodology, Formal analysis, Data curation, Conceptualization. **Leny Pearman:** Writing – review & editing, Data curation. **Yamei Wang:** Writing – review & editing, Formal analysis, Data curation, Conceptualization. **John I. Boxberger:** Writing – review & editing, Writing – original draft, Data curation, Conceptualization. **Mathias P. Bostrom:** Writing – review & editing, Data curation, Conceptualization.

## Declaration of Generative AI and AI-assisted technologies in the writing process

Artificial intelligence (AI) technologies such as Language Learning Models, chatbots, and image creators were not used in the production of this work.

## Role of the funding source

Funding for this post hoc study was provided by Radius Health Inc. (Radius). The sponsor provided financial support to conduct the research and prepare the article. Authors who work for the sponsor participated in the study design, analysis and interpretation of data, in the writing of the report, and in the decision to submit the article for publication.

## Declaration of competing interest

**Neil P. Sheth** reports being a consultant for Medacta, Smith and Nephew, and Zimmer Biomet, and a board/committee member for AAOS, Anterior Hip Foundation, Arab Health, Eastern Orthopaedic Association, and OrthoInfo, and editorial/governing board member for the Journal of Arthroplasty, JBJS-AM, CORR, JAAOS, Journal of Hip Surgery, Journal of Knee Surgery, and the Indian Arthroplasty Journal.

**Renaud Winzenrieth** is a former employee of 3D-Shaper, a paid consultant to Radius Health Inc. (Radius).

**Ludovic Humbert** is an employee and stockholder of 3D-Shaper, a paid consultant to Radius.

**Jared Torkelson** received funding from Radius for travel expenses to present a related congress poster, board member/president of the American Society of Osteoporosis Providers.

**Yamei Wang**, **Leny Pearman**, and **John Boxberger** are employees of Radius.

**Kelly D. Krohn** reports being a presenter for Radius.

**Mathias Bostrom** reports being a consultant for Smith and Nephew, an editorial/governing board member for HSS Journal, a board member for Hip Society and American Austrian Foundation, ownership interest in nonpublic entity HS^2, and royalties from Smith and Nephew.

## Data Availability

Data that underlie the results reported in a published article may be requested for further research 6 months after completion of FDA or EMA regulatory review of a marketing application (if applicable) or 18 months after trial completion (whichever is latest). Radius Health, Inc. will review requests individually to determine whether (i) the requests are legitimate and relevant and meet sound scientific research principles, and (ii) are within the scope of the participants' informed consent. Prior to making data available, requestors will be required to agree in writing to certain obligations, including without limitation, compliance with applicable privacy and other laws and regulations. Proposals should be directed to info@radiuspharm.com.
